# Is a Constricted Imaginal Capacity a Component of the Alexithymia Construct? A Factor Analytic Exploration in a Clinically Enriched Sample

**DOI:** 10.3390/jcm15103624

**Published:** 2026-05-08

**Authors:** R. Michael Bagby, Paweł Larionow, Cheyenne S. McIntyre, Ardeshir Mortezaei, Sharlane Lau, Tamera Cambridge, Graeme J. Taylor

**Affiliations:** 1 Department of Psychology, University of Toronto, 1265 Military Trail, Toronto, ON M1C 1A4, Canada; tamera.cambridge@alumni.utoronto.ca; 2 Department of Psychiatry, University of Toronto, 250 College St., Toronto, ON M5T 1R8, Canada; graeme.taylor@utoronto.ca; 3 Faculty of Psychology, University College of Professional Education, 53-329 Wrocław, Poland; larionow.research@gmail.com; 4 Department of Psychological Science, Ball State University, Muncie, IN 47306, USA; cheyenne.mcintyre@bsu.edu; 5 Department of Psychology, D’Youville University, Buffalo, NY 14201, USA; ardeshir.mortezaei@ontariotechu.net; 6 The Graduate Center, City University of New York, New York, NY 10017, USA; clau@gradcenter.cuny.edu

**Keywords:** alexithymia, imaginal processes, daydreaming, factor analysis, *pensée opératoire*, default mode network, analytic space, transdiagnostic

## Abstract

**Background/Objectives**: Alexithymia is a potential transdiagnostic risk factor for psychiatric and medical disorders involving affect dysregulation. But there is debate over whether a constricted imaginal capacity constitutes a core component of the construct or is a separable correlate. Our aim was to further explore this controversy as any revision to the construct has implications for clinical formulation, neurobiological models of associated default mode network dysfunction, and the coherence of the original psychiatric conceptualization. **Methods**: To replicate and extend previous factor analytic work, we conducted a two-stage exploratory factor analysis in a community sample that included individuals with a psychiatric history (*N* = 681). Using most of the subscale indicators employed in two prior factor analytic studies, we used a two-stage procedure: Stage 1 re-employed the general design of those studies. Stage 2 re-specified the analytic space by removing emotional reactivity and fantasizing-for-regulation variables that form their own separable latent dimensions. **Results**: Stage 1 yielded a conceptual replication of structural separation between alexithymia and imaginal processes. Stage 2 revealed meaningful cross-loadings obscured in the full set, most notably positive constructive daydreaming loading negatively on an externally oriented thinking factor, providing empirical support for the *pensée opératoire* construct. Additional cross-loadings linked dysphoric daydreaming and poor attentional control with negative emotion appraisal. **Conclusions**: The structural independence of alexithymia and imaginal processes is partly contingent on indicator set composition. Construct revision should be grounded not on two factor analytic studies using abbreviated self-report measures, but in convergent evidence across methods, including neuroimaging, electroencephalography, structured interview, performance-based assessment, and clinical observation.

## 1. Introduction

### 1.1. Alexithymia as a Transdiagnostic Construct

Alexithymia is a multifaceted personality construct characterized by difficulties identifying one’s own emotional feelings and distinguishing between feelings and bodily sensations of emotional arousal, difficulty describing feelings to others, and an externally oriented thinking style accompanied by a constricted imaginal capacity [1,2]. This conceptualization of the construct is sometimes referred to as the original, affect deficit model [3] and converges on four inter-related core components: difficulty identifying feelings (DIF), difficulty describing feelings (DDF), externally oriented thinking (EOT), and impoverished imaginal processes (IMP) [2,4]. Initially observed among patients with so-called “classic” psychosomatic diseases and later among individuals with posttraumatic and substance use presentations [1,5,6,7], alexithymia has since been associated with a wide range of psychiatric disorders, including depression, anxiety, eating disorders, somatic symptom disorders, and personality disorders [2,8,9]. Alexithymia, at least as conceptualized according to the original, affect deficit model, is also considered one of several possible risk factors for a variety of physical illnesses, including cardiovascular disease, functional gastrointestinal disorders, diabetes, and chronic pain, and it can potentially influence illness behavior and impact treatment outcomes [10,11]. Alexithymia has accordingly been proposed as a transdiagnostic factor increasing vulnerability for emotion-based psychopathology [4,12] and for some medical disorders [10].

### 1.2. Alexithymia and the Constricted Imaginal Capacity Component

Although the name “alexithymia” was first coined by Sifneos [6], the clinical observations underlying it predated the term [13,14]. Subsequent formulations articulated alexithymia as encompassing DIF, DDF, EOT, and a constricted imaginal capacity [2]. Critically, imaginal impoverishment was not defined simply as low daydreaming frequency but as reduced symbolic richness, limited imaginative elaboration, and diminished capacity for internally generated affective experience [1,5].

The conceptual relationship between EOT and constricted imaginal processes has roots in the French psychosomatic medicine tradition. Marty and de M’Uzan [15] introduced the term *pensée opératoire* (operational thinking) to describe a cognitive style characterized by concrete, stimulus-bound, utilitarian thought with a conspicuous absence of symbolic elaboration and fantasy. In their formulation, the paucity of imaginal life and the externally oriented cognitive style were two manifestations of the same disturbance in mental representation. Taylor et al. [16] recently revisited this relationship, arguing that EOT and constricted imaginal processes reflect overlapping facets of diminished symbolic elaboration, predicting structural permeability between EOT and elaborative fantasizing indicators. Under the *pensée opératoire* formulation, the most informative test is not whether indicators of fantasizing form a separate factor, but whether elaborative fantasizing shows an inverse structural relationship with EOT.

### 1.3. The Current Debate: Proposals to Remove the Impoverished Imaginal Processes Component

The most widely used operationalization of alexithymia is the 20-item Toronto Alexithymia Scale (TAS-20) [17,18], which assesses DIF, DDF, and EOT [19]. Although the TAS-20 does not include a subscale that directly assesses fantasizing and other imaginal activity, it was developed within a theoretical framework in which constricted imaginal processes were considered central. Fantasizing is thought to be indirectly captured by items on the EOT subscale [19]. The capacity for imagination is also assessed with a subscale of the Toronto Structured Interview for Alexithymia (TSIA) [20] and with performance-based tests [21,22,23].

Despite this long theoretical history, there have been recent calls to remove the imaginal processes component from the alexithymia construct, resting primarily on two factor analytic investigations by Preece and colleagues in non-clinical samples. Preece et al. [12] jointly factor analyzed the subscales of the TAS-20 and the Perth Alexithymia Questionnaire (PAQ [24]) alongside indicators of fantasizing, daydreaming, and emotional reactivity. The authors reported structural separation and proposed that the results supported a three-component “attention appraisal” model of alexithymia that excludes imaginal processes. Preece and Gross [25] repeated this analysis using the same imaginal process measures and reported a five-factor solution in which alexithymia and imaginal process indicators were clearly separable. Based on these two studies, it was concluded that imaginal processes are not a salient component of the alexithymia construct.

### 1.4. Analytic Space and the Limits of Factor Analytic Inference

While the statistical results from Preece and colleagues’ factor analytic investigations [12,25] are correctly interpreted, the method of factor analysis can only adjudicate covariance among indicators within a defined *analytic space*, or the set of indicators jointly entered into the factor model. Adding indicators that form their own separable latent dimensions can partition covariance among remaining indicators, changing apparent construct boundaries. Latent structure results are therefore contingent upon the selection and representation of variables included in the model. Demonstrating psychometric separability establishes distinguishability under specific operationalizations but does not determine whether an impoverished imaginal capacity as classically described is conceptually extrinsic to alexithymia.

Preliminary evidence for this analytic contingency argument emerged in a study by Larionow et al. [26], whose subscale-level exploratory factor analysis (EFA) of the TAS-20, the Emotion Regulation Questionnaire-Fantasizing (ERQ-F [12]), the Patient Health Questionnaire-4 (PHQ-4 [27]), and the World Health Organization-Five Well-Being Index (WHO-5) [28] indicators in a Polish community sample (*N* = 271) yielded three factors—alexithymia, fantasizing for emotion regulation, and psychological distress—with the ERQ-F loading exclusively on its own factor. These findings illustrate that when alexithymia is factor analyzed alongside variables that reliably form their own latent dimensions—the ERQ-F and the PHQ-4/WHO-5 subscales each constituting separate factors—the resulting structure partitions covariance between alexithymia and its correlates, producing a cleaner factorial separation than the inter-factor correlations would suggest.

A related methodological concern involves indicator density. In the study by Preece et al. [12], 17 non-alexithymia indicators were entered alongside eight alexithymia indicators; the imbalance in Preece and Gross’s study [25] was similar. Factors defined by more indicators extract more variance and emerge as more clearly differentiated, while underrepresented domains may have their covariance partitioned across other factors [29,30]. This imbalance does not invalidate the results but constrains their interpretation—structural separation under such conditions is not equivalent to separation under balanced representation.

A further methodological concern involves two of the measures created specifically for these investigations. The Imaginal Processes Inventory-45 (IPI-45) used by Preece and colleagues [12,25] was an unpublished, post hoc adaptation derived by selecting five items per subscale from factor loadings reported by Giambra [31] for the 344-item version of the IPI [32]. This adaptation was validated via confirmatory factor analysis in the same sample used for the primary EFA, which poses a significant capitalization-on-chance concern that the authors acknowledged [12] but did not weigh against their inferential claim. The resulting nine-subscale structure contributed nine of the 25 indicators in the factor space, compounding the indicator imbalance problem described above. In the present study, we replaced this measure with the Short Imaginal Processes Inventory (SIPI [33,34,35]), which has an established psychometric record, and a validated three-factor structure whose subscales (Positive-Constructive Daydreaming, Guilt and Fear-of-Failure Daydreaming, Poor Attentional Control) map onto qualitatively distinct daydreaming styles rather than fragmenting the domain into narrow content themes [36]. The ERQ-F was also purpose-built for these studies [12], designed to measure the use of fantasy as a deliberate emotion regulation strategy, a variable conceptually aligned with the attention appraisal model emphasis on emotion regulation rather than with the original affect deficit model emphasis on representational capacity. The separation of fantasizing for emotion regulation from alexithymia in factor analysis is therefore partially expected by design. This does not invalidate the finding, but it does constrain the inferential weight that should be placed on the ERQ-F’s factorial independence from the alexithymia factor.

### 1.5. Imaginal Processes and the Neuroscience of Internally Generated Cognition

The status of imaginal processes within alexithymia is also consequential from a medical/neuroscience perspective. Imagination, understood as constructive simulation and recombination of internally generated neural representations depends on coordinated activity within large-scale neural systems, particularly the default mode network (DMN), which supports spontaneous thought, the generation and manipulation of mental images, daydreaming, mind-wandering, introspection/autobiographical construction, and self-referential processing [37,38,39,40,41,42]. Neuroimaging and electroencephalographic (EEG) studies have documented DMN alterations in alexithymia, including reduced posterior cingulate cortex activation during imagery tasks [43] and diminished resting-state connectivity within the DMN [44,45]. In one EEG study, the DDF and EOT subscales of the TAS-20 correlated negatively with alpha-band connectivity between the right anterior cingulate cortex and the right posterior cingulate cortex [45]. These findings suggest that alexithymia involves disrupted activity and/or connectivity in neural networks for precisely the kind of internally generated, elaborative cognition that constitutes imaginal processes. There is also some preliminary evidence that the externally oriented cognitive style that characterizes *pensée opératoire* maps neurobiologically onto reduced engagement of DMN regions supporting internally generated, self-referential thought [37]. Moreover, there is an anticorrelation between the DMN and the dorsal attention network (DAN) so that when the focus of attention is on the external world, there is a suppression of spontaneous internal cognitive processes including remembering past experiences and imagining the future [37,46]. This convergence between clinical observation, neuroimaging and EEG evidence, and the classical psychiatric formulation underscores why the question of whether the constricted imaginal processes component belongs within the alexithymia construct cannot be adjudicated by factor analysis alone.

### 1.6. Clinical Implications of the Structural Question

The question concerning the IMP component of the alexithymia construct has direct implications for clinical psychiatry. If imaginal impairment is separable from alexithymia, clinical assessment may justifiably focus on affect identification while treating imaginal capacity as an independent domain. Conversely, if a limited imaginal capacity is structurally intertwined with EOT, consistent with the classical *pensée opératoire* formulation, then the clinical phenotype of alexithymia may be broader than currently operationalized in dominant self-report measures of the construct. For clinicians, misattributing variance across affect labeling, cognitive style rigidity, and impaired symbolic capacity could influence both diagnosis and treatment planning.

### 1.7. The Present Study

A critical conceptual distinction must be drawn before specifying the study aims. The classical clinical formulation of constricted imaginal processes encompasses at least two levels. The first is a *quantitative behavioral level* concerning the frequency and content valence of the fantasizing and daydreaming dimensions assessable by existing self-report measures such as the Daydreaming Frequency Scale (DFS [47]) and SIPI [34]. The second is a *qualitative symbolic level* concerning the depth, vividness, symbolic elaboration, and emotional differentiation of fantasy life, the dimension originally described by Nemiah et al. [1], elaborated by Krystal [5], and recently revisited by Taylor et al. [16]. Available self-report measures such as the TAS-20 and the PAQ operate at the first level; they do not directly assess the qualitative impoverishment of symbolic fantasy that is central to the clinical construct. The present analyses therefore evaluate the structural relationship between alexithymia and imaginal processes as operationalized by quantitative behavioral indicators. This is an informative but necessarily incomplete proxy, and conclusions about structural separation or permeability are constrained accordingly.

The study had two aims and was conducted in two stages. In the first stage, we sought to replicate conceptually the Preece et al. [12] and Preece and Gross [25] EFA in a larger and more heterogeneous sample using improved imaginal process measures. We hypothesized (H1) that in the full indicator set, including the ERQ-F and the Perth Emotional Reactivity Scale-Short Form (PERS-S [48]) as well as alexithymia and imaginal process measures, alexithymia and imaginal process indicators would form separable factors, and thereby replicate prior investigations (i.e., [12,25]). In the second stage, our aim was to examine what happens to the latent structure when the analytic space is re-specified by removing the ERQ-F and PERS-S indicators. We hypothesized (H2) that in the reduced indicator set, imaginal process indicators would show meaningful cross-loadings with alexithymia factors—specifically, Positive-Constructive Daydreaming with EOT (consistent with *pensée opératoire*), and Guilt and Fear-of-Failure Daydreaming and Poor Attentional Control with negative emotion appraisal—and thereby indicate boundary permeability obscured in the full-set analysis. If H1 is supported but H2 is not, this would provide robust evidence for structural distinction regardless of analytic configuration. If both are supported, separation is partly contingent on indicator set composition. By clarifying the structural boundaries of alexithymia, the findings aim to inform clinical assessment interpretation and contribute to ongoing debates regarding the cognitive architecture underlying affect dysregulation.

## 2. Materials and Methods

### 2.1. Participants

Participants were 681 adults (63.7% female; *M* age = 36.1, *SD* = 13.8) from a clinically enriched community sample recruited through the Prolific platform, London, UK. The sample included participants who reported a history of psychiatric diagnosis (28.2%) or psychiatric or psychological treatment (39.9%). Primary diagnoses reported included depression, anxiety disorders, posttraumatic stress disorder, personality disorders, and substance use disorders. Some data from these participants were reported in a separate study with different aims and analytic methods [49]; none of those data overlap with the present analyses. More details about the sample are provided in that study or available upon request from the first author.

To enhance external validity, recruitment parameters were approved by the Research Ethics Board (REB) at the University of Toronto and intentionally retained individuals reporting past or current psychiatric diagnoses or treatment, thereby enriching the sample for psychiatric symptomatology while approximating outpatient clinical heterogeneity.

### 2.2. Measures

*Alexithymia.* The TAS-20 [17,18] comprises three subscales—DIF (7 items), DDF (5 items), and EOT (8 items)—rated on a 5-point Likert scale. This instrument has proven to be reliable and valid; for a comprehensive review, see Bagby et al. [19]. The PAQ [24] comprises five subscales assessing valence-specific emotion identification and description difficulties (Negative-DIF, Positive-DIF, Negative-DDF, Positive-DDF; 4 items each) and general externally oriented thinking (G-EOT; 8 items), rated on a 7-point Likert scale. This instrument has evidence of reliability and validity [24].

*Imaginal processes.* The Daydreaming Frequency Scale (DFS [47]) is a 12-item measure of general daydreaming frequency. Three subscales of the SIPI [33,34] assessed qualitative dimensions of daydreaming: Positive-Constructive Daydreaming (PCD; 15 items), Guilt and Fear-of-Failure Daydreaming (GFF; 15 items), and Poor Attentional Control (PAC; 15 items).

*Fantasizing for emotion regulation.* The Emotion Regulation Questionnaire–Fantasizing (ERQ-F; 6 items [12]).

*Emotional reactivity.* The Perth Emotional Reactivity Scale–Short Form (PERS-S [48]), comprising six subscales crossing two valence dimensions (Negative, Positive) with three temporal features (Activation, Intensity, and Duration; 3 items each).

### 2.3. Analytic Approach

Analyses were conducted in JASP 0.19.3, with missing data (<0.5%) input using the *jYS* module in *JAMOVI* 2.7.17, University of Melbourne, Australia. For the purpose of an attempt to replicate conceptually Preece and colleagues’ previous factor analytic investigations, we employed their same statistical procedures; all EFAs used principal axis factoring (PAF) with direct oblimin rotation, factor retention was determined by parallel analysis (PA) using Pearson correlation matrices. A standardized factor loading of ≥0.40 was considered salient and interpretable, and cross-loadings ≥ 0.30 were considered marginally meaningful and reported [50]. To determine if a matrix was suitable for PAF we applied the Kaiser–Meyer–Olkin (KMO) and Bartlett tests. Pattern matrix loading coefficients are reported throughout. Because direct oblimin is an oblique rotation, the analysis also yields a factor correlation matrix; inter-factor correlations reported in the text are drawn from these matrices. Analyses proceeded in two stages.

In keeping with the analytic strategy of Preece et al. [12] and Preece and Gross [25], the EFAs were conducted at the subscale level rather than the item level. This level of analysis is consistent with the structural question of the study, which concerns whether alexithymia and imaginal process constructs form separable latent dimensions. The subscales of the instruments employed here were developed and validated to function as coherent indicators of their target constructs; they aggregate item-level variance and attenuate item-specific method variance and content idiosyncrasy that would otherwise complicate construct-level structural inference. The participant-to-subscale ratio in Stage 1 (35.84:1) and Stage 2 (56.75:1) substantially exceeds conventional standards for factor analysis [50]. Factorability was therefore evaluated at the level of analysis conducted, using the Kaiser–Meyer–Olkin and Bartlett tests applied to the subscale matrix in each stage rather than to an item-level correlation matrix.

*Stage 1* attempted to replicate the Preece et al. [12] and Preece and Gross [25] EFA using 19 of their 25 indicators (TAS-20, PAQ, DFS, SIPI, ERQ-F, and PERS-S) (we included the three subscales of the SIPI rather than the nine subscales of the IPI-45 used by Preece and colleagues). Replication was defined operationally as convergence in the number of factors retained by parallel analysis, the substantive clustering of indicators onto factors (i.e., mostly the same indicators defining the same latent dimensions), and the interpretability of factor labels relative to those reported by Preece and Gross [25]. This criterion permits assessment of structural correspondence despite differences in the SIPI and the IPI-45.

*Stage 2* re-specified the analytic space by removing the ERQ-F and all six PERS-S subscales, reducing the analysis to 12 indicators of alexithymia and daydreaming. The rationale for this re-specification is detailed in Section 1.4; because the ERQ-F and PERS-S subscales consistently emerge as separable latent dimensions [12,25,26], their inclusion in Stage 1 may partition covariance between the constructs of primary interest. Stage 2 tests whether the alexithymia–imaginal process boundary remains stable when these indicators are removed.

## 3. Results

### 3.1. Preliminary Analyses

Descriptive statistics and reliability coefficients for all the study measures are presented in Table 1. Internal consistency was acceptable for all subscale and total scores (ω and α ≥ 0.79). The exception was the EOT subscale of the TAS-20 (ω = 0.63, α = 0.64), which was marginal but consistent with the well-documented multidimensionality of this subscale [19,51]. Any loading involving EOT is therefore attenuated by measurement error, meaning observed associations likely underestimate the true structural overlap. The distributional properties of all measures approximated the normal distribution and were neither skewed nor kurtotic.

### 3.2. Bivariate Associations

Pearson correlations among study measures are presented in Table 2. Positive-Constructive Daydreaming (PCD), which measures the elaborative, rich imaginal activity whose absence defines impoverished fantasy in the original formulation of alexithymia, was negatively correlated with the TAS-20 EOT score (*r* = –0.31), total score (*r* = –0.20), and PAQ EOT score (*r* = −0.26) and total score (*r* = –0.19). These small but clinically meaningful associations [52], particularly with EOT, are consistent with the construct of *pensée opératoire*. In contrast, Guilt and Fear-of-Failure Daydreaming (GFF) showed moderate but positive correlations with TAS-20 DIF (*r* = 0.42), DDF (*r* = 0.35), and total TAS-20 scores (*r* = 0.37), and Poor Attentional Control (PAC) showed a similar magnitude, but positive association (TAS-20 DIF: *r* = 0.36; total: *r* = 0.33). The GFF and PAC scales also showed moderate positive correlations with the positive and negative valence DIF and DDF subscales of the PAQ and with its total score. Among all correlations between EOT subscales and imaginal process indicators, the largest was between TAS-20 EOT and PCD (*r* = –0.31). Thus, higher levels of alexithymia are associated with less positive constructive daydreaming but more guilt and fear-of-failure daydreaming and poor attentional control. The ERQ-F was weakly correlated with alexithymia measures (*r* = 0.08–0.14) but substantially correlated with DFS (*r* = 0.56) and PCD (*r* = 0.62), confirming at the bivariate level what Larionow et al. [26] demonstrated factorially: the ERQ-F is more strongly aligned with imaginal process indicators than with alexithymia.

### 3.3. Stage 1: Full Measurement Set Exploratory Factor Analysis (Replication)

The KMO test (0.87) and Bartlett’s test (χ^2^(171) = 10,402.07, *p* < 0.001) confirmed factorability. Parallel analysis indicated five factors. Table 3 displays the rotated pattern matrix; factor intercorrelations appear in Table 4. Consistent with H1, this solution represents a conceptual replication of the core structural finding reported by Preece et al. [12] and Preece and Gross [25]. Five factors emerged from the extraction procedure, four of which corresponded to those reported by Preece and colleagues, including an alexithymia factor (Factor 1), negative and positive emotional reactivity factors (Factors 2 and 3, respectively), and a general daydreaming factor (Factor 4).

While Preece and Gross [25] reported a separate factor for negative emotional daydreaming, this factor did not emerge in our analyses, likely because the SIPI contains a single negative daydreaming subscale whereas the IPI-45 included three. Additionally, in our sample an EOT factor emerged with the TAS-20 and PAQ EOT subscales exhibiting salient loadings on Factor 5. Of note is that the PAQ G-EOT also had a salient cross-loading on Factor 1 (λ = 0.42).

These differences do not erode H1, as emotional reactivity and general daydreaming loaded on separate factors without cross-loading on the alexithymia factor, conceptually replicating the findings of Preece and colleagues [12,25]. Within this frame of overall replication several other notable patterns did emerge from these results; while Factor 2 was defined by the three PERS-S negative reactivity subscales, it did have secondary loadings from GFF (λ = 0.30) and PAC (λ = 0.41), which is not consistent with the suggestion of Bermond et al. [53] that impoverished imaginal processes are associated with reduced emotional reactivity (if it were, the loading would have been negative). The high loading of ERQ-F on the general daydreaming factor (λ = 0.77), despite near-zero correlations with alexithymia (*r* = 0.10 for TAS-20 total and *r* = 0.08 for PAQ total), effectively anchors that factor around regulatory fantasy and general daydreaming frequency, pulling it away from the alexithymia domain.

### 3.4. Stage 2: Reduced Measurement Set Exploratory Factor Analysis (Respecified)

Removing the ERQ-F and all six PERS-S subscales reduced the indicator set to 12 variables. The KMO test (0.85) and Bartlett’s test (χ^2^(66) = 6424.60, *p* < 0.001) confirmed factorability. Parallel analysis indicated four factors, explaining 68.98% of total variance. Table 5 displays the rotated pattern matrix; factor intercorrelations appear in Table 6. Factor 1 represented negative emotion appraisal difficulty (TAS-20 DIF: λ = 0.90; DDF: λ = 0.62; PAQ N-DIF: λ = 0.77; PAQ N-DDF: λ = 0.57). Factor 2 represented positive emotion appraisal difficulty (PAQ P-DIF: λ = 0.77; PAQ P-DDF: λ = 0.89). Factors 1 and 2 were highly correlated (*r* = 0.79), likely reflecting valence-specific method structure inherent to the PAQ rather than a substantive distinction. Factor 3 was an imaginal processes factor (DFS: λ = 0.93; PCD: λ = 0.49; GFF: λ = 0.37; PAC: λ = 0.54). Factor 4 was an EOT factor (TAS-20 EOT: λ = 0.72; PAQ G-EOT: λ = 0.62).

Consistent with H2, three meaningful cross-loadings emerged that were absent or attenuated in the full-set analysis, each corresponding to a theoretically predicted pattern of boundary permeability:

*Dysphoric daydreaming and negative appraisal.* Guilt and Fear-of-Failure Daydreaming loaded both on the imaginal factor (λ = 0.37) and on the negative emotion appraisal factor (λ = 0.40), indicating dual membership; this form of dysphoric daydreaming is simultaneously an imaginal activity and a correlate of difficulty processing negative emotions. The high correlation between the two appraisal factors (*r* = 0.79) raises the possibility that valence-shared content contributes to this cross-loading.

*Attentional dyscontrol and negative appraisal.* Poor Attentional Control loaded primarily on the imaginal factor (λ = 0.54) but cross-loaded (λ = 0.36) on negative emotion appraisal, linking attentional dysregulation during daydreaming to broader difficulties with negative affect awareness.

*Constructive daydreaming and externally oriented thinking (pensée opératoire).* Of particular theoretical importance, Positive-Constructive Daydreaming loaded (λ = 0.49) on the imaginal factor but cross-loaded (λ = –0.31) on the EOT factor, consistent with the concept of *pensée opératoire* [15,16]. This cross-loading meets the marginal threshold established a priori, and the true value is likely attenuated by the low reliability of EOT (ω = 0.63); nonetheless, it should be interpreted with appropriate caution given its magnitude. The association was masked in the full-set analysis (Stage 1), where the ERQ-F and PERS-S indicators absorbed the relevant covariance.

As a sensitivity check, both EFAs were re-estimated using polychoric correlation matrices. The factor retention decisions, primary loading patterns, and the three critical Stage 2 cross-loadings were materially unchanged, confirming that the results are not an artifact of treating Likert-derived subscale scores as continuous.

## 4. Discussion

The present study conceptually replicated and extended the finding by Preece et al. [12] and Preece and Gross [25] that alexithymia and imaginal process indicators form structurally separable latent dimensions when analyzed in a broad indicator set including emotional reactivity and fantasizing-for-regulation measures. This conceptual replication was achieved in a larger, more heterogeneous sample enriched with psychiatric history, using psychometrically validated imaginal process measures in place of the unpublished IPI adaptation used in the prior studies. The five-factor solution from Stage 1 can now be treated as a replicable structural finding under broad analytic specification.

At the same time, our results demonstrate that this structural separation is partly contingent on the composition of the analytic space. When the indicator set was restricted to the constructs most directly relevant to the alexithymia–imaginal process debate (i.e., removing the ERQ-F and PERS-S indicators that reliably form their own latent dimensions), meaningful cross-loadings emerged that had been obscured in the full-set analysis. The interpretive significance of psychometric separability therefore depends critically on what else is in the analytic space. This is not a statistical artifact but a theoretically important demonstration—adding indicators that form separable latent dimensions partitions covariance among remaining indicators and can make construct boundaries appear sharper than they are under more focused specification. This point applies to any investigation where variables with strong own-factor allegiance absorb shared variance between constructs of primary interest. The converse concern also applies reducing from 25 to 12 indicators could force indicators onto fewer factors. However, the pattern of cross-loadings corresponds to theoretically predicted patterns rather than an arbitrary statistical compression, and their convergence with bivariate correlations and clinical reports argues against a purely artifactual interpretation of these results.

The Positive-Constructive Daydreaming subscale cross-loading on the EOT factor warrants attention as the most theoretically informative result. Critically, this cross-loading emerged despite the low internal reliability of the TAS-20 EOT subscale (ω = 0.63), which attenuates all loadings involving this factor; the fact that a theoretically predicted structural relationship is detectable even with an indicator with low internal reliability suggests the true association is likely stronger than we found. The magnitude is modest and at the marginal threshold, yet this pattern provides empirical support for the concept of *pensée opératoire* [15,16]—individuals high on EOT report less positive constructive daydreaming, which is precisely the structural permeability predicted by the classical formulation. If EOT and imaginal processes were truly orthogonal constructs, as the Preece and Gross [25] account implies, this cross-loading, and its convergence with both the clinical tradition and evidence from non-self-report measures of alexithymia, would be difficult to explain. The bivariate analysis provides additional context as the negative correlation between TAS-20 EOT and PCD (*r* = −0.31) reveals a moderate effect size association, which is consistent with the factor analytic results. To contextualize the magnitude of this association, correcting the bivariate correlation for attenuation due to the low reliability of the TAS-20 EOT indicator (ω = 0.63) yields an estimated true-score correlation of approximately *r* = −0.42, suggesting that the observed cross-loading underestimates the structural relationship by a non-trivial margin.

Taken together, Stage 1 confirmed structural separation under broad analytic specification, and Stage 2 revealed boundary permeability under focused specification, with three theoretically coherent cross-loadings emerging once the ERQ-F and PERS-S indicators were removed. The high inter-factor correlation (*r* = 0.79; Table 6) between the negative and positive emotion appraisal factors in Stage 2 likely reflects the valence architecture of the PAQ rather than a substantive distinction between two appraisal processes. Factor 1 combines the TAS-20 DIF and DDF subscales, whose item content skews toward negative emotional experience, with the PAQ negative valence subscales, while Factor 2 is defined exclusively by the two PAQ positive-valence subscales. Bagby et al. [54], using exploratory sequential hierarchical factoring, demonstrated that the PAQ does not decompose into its proposed five-factor dimensional structure but instead measures a single, unidimensional construct, with the differentiation between positive and negative emotion facets being empirically weak. Consistent with this interpretation, Zahid et al. [55] found that the PAQ’s valence-specific DIF and DDF subscales failed to demonstrate incremental validity over the TAS-20 in predicting two constructs theoretically related to alexithymia, indicating that the positive–negative emotion distinction does not capture empirically distinct variance. The factor separation observed here is thus consistent with valence-specific method variance within a single instrument rather than with two genuinely distinct appraisal constructs. Critically, the cross-loadings central to the present argument are not dependent on this valence split as the PCD cross-loading resides on the EOT factor, which contains no PAQ valence-specific content, and the GFF and PAC cross-loadings fall on the negative appraisal factor, which includes both TAS-20 and PAQ indicators. To ensure that the critical cross-loadings were not an artifact of over-extracting valence-specific method factors, we examined a three-factor solution collapsing the two appraisal factors into a single emotion appraisal dimension. In this analysis, the PCD cross-loaded on the EOT factor (λ = −0.31), the GFF cross-loaded on the emotion appraisal factor (λ = 0.35); the PAC cross-loading on emotion appraisal, however, was trivial (λ = −0.07). Overall, this pattern confirms that the Stage 2 findings are robust to this specification and consistent with our H2 hypothesis.

### 4.1. Clinical Implications

The present findings carry several implications for clinical assessment and formulation. First, cross-loadings between positive constructive daydreaming and externally oriented thinking suggest these may not be fully independent domains, aligning with *pensée opératoire*. When self-report instruments isolate affect identification (DIF/DDF) from cognitive style (EOT), clinicians may underappreciate the potential role of imaginal constriction in patients presenting with rigid, externally focused thinking. In practical terms, when a clinician observes an elevated EOT score on the TAS-20, the present findings suggest it may be informative to evaluate the quality of the patient’s imaginal capacity using interview-based methods such as the TSIA [20] or performance-based approaches [21,22,23], rather than relying solely on the self-report profile. Nonetheless, there is evidence that the TAS-20 EOT subscale is associated negatively, and the PCD subscale of the SIPI positively, with the Openness to Experience domain of the five-factor model of personality [56,57], which is consistent with the view that individuals with high levels of alexithymia have limited ability to experience and enjoy playful and imaginative fantasies, and lack curiosity and interest in exploring ideas, feelings, and creative endeavors. The cross-loading of the PAC subscale on Factor 1 suggests that individuals who are poor at identifying and describing affects are unable to focus attention on internal thought and sustain elaborate fantasies and may be prone to boredom. The cross-loading of GFF on Factor 1 is consistent with the dysphoric quality of the fantasies and with evidence that this subscale of the SIPI is associated with the Neuroticism domain of the five-factor model of personality [57], as also are the DIF and DDF subscales of the TAS-20 [56].

Second, the findings caution against over-interpreting structural independence in multivariate models that include extensive appraisal measures. If appraisal-related variance dominates the factor space, structural conclusions may reflect methodological configuration rather than psychological architecture.

Third, if externally oriented thinking is structurally linked with diminished constructive imagery, interventions targeting symbolic elaboration or imagery-based processing may engage mechanisms that extend beyond affect labeling alone. Indeed, both Singer [58] and Taylor [59] have argued that helping patients to focus on internal images and experience constructive symbolic fantasies generates the positive affect of interest [60], which thereby mitigates negative affects.

Finally, if imaginal constriction and externally oriented thinking are partially intertwined, neuroimaging and EEG findings attributed solely to emotion identification deficits may reflect broader cognitive symbolic architecture, with implications for interpreting both clinical and neuroscientific research on alexithymia.

### 4.2. Construct Revision Requires Convergent Evidence

The proposal to remove the imaginal processes component from the alexithymia construct rests, at present, on two factor analytic studies from the same research group using self-report measures in non-clinical samples. We do not question the internal validity of those studies, but question whether two EFA investigations provide a sufficient evidentiary basis for revising a construct with more than fifty years of clinical and empirical lineage. The available evidence from other methods points in the opposite direction: neuroimaging and EEG studies document DMN and other neural network alterations in alexithymia that implicate precisely the neural systems supporting internally generated, elaborative cognition [43,44,45,61]; clinical observation continues to identify impoverished fantasizing as a salient feature of alexithymic presentation [16]; structured interview methods such as the TSIA [20] assess imaginal impoverishment directly; and performance-based approaches, including Rorschach indices of alexithymia [21,22], provide convergent evidence of impoverished imaginal capacity that bypasses the limitations of self-report scales “that ask people to report retrospectively on longstanding patterns of mentation” ([62] p. 732). Factor analytic separability between abbreviated self-report operationalizations does not override this convergent evidence, particularly when the factor analytic results themselves are shown to be contingent on analytic specification.

We close with what we consider the most consequential point. The imaginal process measures available for this type of analysis assess daydreaming frequency, content valence, attentional control, and regulatory use of fantasy—the quantitative behavioral level—but not the qualitative impoverishment of symbolic elaboration and imaginative richness originally described in the clinical literature [1,5,15,16]. This measurement gap constrains all factor analytic investigations of this question, including those by Preece and colleagues and the present study. Both the conclusion that imaginal processes are structurally distinct, and the present finding of boundary permeability are drawn from the same incomplete measurement space. Until measures capable of assessing the qualitative-symbolic dimension are incorporated into the factor analytic space, the structural relationship between alexithymia and constricted imaginal processes cannot be definitively evaluated.

### 4.3. Limitations

Several limitations should be noted. The study relied on a single cross-sectional sample recruited through an online platform, and the EFA results were not confirmed through confirmatory factor analysis in an independent sample. This limitation is particularly relevant given that the present study critiques Preece and colleagues [12] use of same-sample CFA validation for the IPI-45; the same standard applies to our own results and represents an important direction for future replication. Although the sample was enriched with psychiatric history, replication in clinical populations and in samples with greater cultural and socioeconomic diversity would strengthen generalizability. Additionally, the indicator imbalance we identify in the Preece et al. [12] and Preece and Gross [25] studies apply in modified form to our Stage 2, where eight alexithymia indicators were entered alongside four imaginal process indicators. Importantly, however, the two imbalances carry asymmetric consequences: Preece and colleagues’ overrepresentation of non-alexithymia indicators strengthens those factors and suppresses cross-loadings—the very conclusion drawn—whereas our reversed ratio could facilitate cross-loadings from under-represented imaginal indicators onto alexithymia factors, which is also the direction that will detect boundary permeability if it exists. The PCD cross-loading on EOT is least vulnerable to this concern, as the EOT factor is defined by only two indicators. The theoretical coherence of the emergent cross-loadings mitigates but does not eliminate this limitation. All measures were self-report, which may not adequately capture the qualitative impoverishment of symbolic fantasy central to the original conceptualization of the construct [4]. The low internal consistency of TAS-20 EOT attenuates all loadings involving this factor, meaning that the true structural overlap between externally oriented thinking and imaginal processes may be stronger than observed estimates suggest. Relatedly, the present analyses, like those of Preece et al. [12] and Preece and Gross [25], were conducted at the subscale rather than the item level. While subscale-level modeling is appropriate for the construct-level structural question addressed here and preserves comparability with the prior studies, an item-level analysis—not undertaken in either the previous studies nor the present one—could detect within-subscale heterogeneity (for example, whether particular TAS-20 EOT items or specific SIPI items contribute disproportionately to the Stage 2 cross-loadings) and would usefully complement the present findings. Future research should incorporate structured interviews, performance-based measures, item-level factor analyses, and confirmatory modeling with preregistered hypotheses to more fully evaluate the structural relationship between alexithymia and imaginal processes.

## 5. Conclusions

The structural separation between alexithymia and imaginal processes is a replicable finding under broad analytic specification, but its interpretation requires qualification. The degree of separation depends on the composition of the analytic space, and meaningful cross-loadings—including one that directly supports the classical concept of *pensée opératoire*—emerge when the space is restricted to the most theoretically relevant indicators. Revisions to a construct with longstanding psychiatric lineage should be grounded not only in latent structure findings derived from abbreviated self-report measures, but in convergent evidence across methods and in the principles of replication and methodological triangulation by which science progresses.

## Figures and Tables

**Table 1:** Descriptive Statistics and Reliability Estimates for All Study Measures.

Variables	McDonald’s Omega (95% CI)	Cronbach’s Alpha (95% CI)	*M*	*SD*	Skew	Kurtosis	Range
TAS-20 DIF	0.91 (0.90; 0.92)	0.91 (0.90; 0.92)	14.41	6.71	0.79	–0.34	7–34
TAS-20 DDF	0.84 (0.82; 0.86)	0.84 (0.82; 0.86)	12.44	5.04	0.42	–0.68	5–25
TAS-20 EOT	0.63 (0.59; 0.67)	0.64 (0.60; 0.68)	18.63	4.68	0.11	–0.44	8–31
TAS-20 Total score	0.90 (0.89; 0.91)	0.89 (0.88; 0.90)	45.48	13.60	0.48	–0.37	20–83
PAQ Negative-DIF	0.92 (0.91; 0.93)	0.92 (0.90; 0.93)	10.64	6.04	0.87	–0.10	4–28
PAQ Positive-DIF	0.92 (0.91; 0.93)	0.92 (0.90; 0.93)	9.60	5.48	1.09	0.57	4–28
PAQ Negative-DDF	0.93 (0.92; 0.94)	0.93 (0.92; 0.94)	12.37	6.70	0.56	–0.77	4–28
PAQ Positive-DDF	0.92 (0.91; 0.93)	0.92 (0.90; 0.93)	11.11	6.00	0.70	–0.40	4–28
PAQ General-EOT	0.92 (0.91; 0.93)	0.92 (0.91; 0.93)	23.56	10.81	0.68	–0.24	8–56
PAQ Total score	0.97 (0.96; 0.97)	0.97 (0.96; 0.97)	67.29	30.75	0.70	–0.19	24–157
DFS Total score	0.95 (0.95; 0.96)	0.95 (0.95; 0.96)	33.94	11.43	0.19	–0.81	12–60
SIPI Positive-Constructive Daydreaming	0.86 (0.84; 0.87)	0.84 (0.82; 0.86)	50.96	10.02	–0.42	0.02	21–73
SIPI Guilt and Fear-of-Failure Daydreaming	0.84 (0.83; 0.86)	0.84 (0.82; 0.86)	31.85	10.06	0.44	–0.32	15–62
SIPI Poor Attentional Control	0.89 (0.87; 0.90)	0.88 (0.87; 0.90)	45.14	11.79	0.01	–0.39	16–75
ERQ-Fantasizing	0.94 (0.93; 0.94)	0.94 (0.93; 0.94)	24.67	9.69	–0.16	–0.84	6–42
PERS-S Negative-activation	0.83 (0.81; 0.85)	0.82 (0.80; 0.84)	8.61	3.41	0.07	–1.04	3–15
PERS-S Negative-intensity	0.85 (0.83; 0.87)	0.85 (0.83; 0.87)	9.48	3.37	–0.05	–0.92	3–15
PERS-S Negative-duration	0.88 (0.86; 0.89)	0.88 (0.86; 0.89)	8.80	3.48	0.06	–1.02	3–15
PERS-S Positive-activation	0.79 (0.77; 0.82)	0.79 (0.76; 0.81)	10.56	2.77	–0.57	–0.11	3–15
PERS-S Positive-intensity	0.87 (0.85; 0.89)	0.87 (0.85; 0.89)	10.95	2.91	–0.74	0.12	3–15
PERS-S Positive-duration	0.87 (0.85; 0.89)	0.87 (0.85; 0.89)	10.65	2.98	–0.72	–0.02	3–15

*Note*: TAS-20 = 20-item Toronto Alexithymia Scale; PAQ = Perth Alexithymia Questionnaire; DFS = Daydreaming Frequency Scale; SIPI= Short Imaginal Processes Inventory; ERQ = Emotion Regulation Questionnaire; PERS-S = Perth Emotional Reactivity Scale-Short Form.

**Table 2:** Pearson correction matrix for the study measures (*N* = 681).

Variables	1	2	3	4	5	6	7	8	9	10	11	12	13	14	15	16	17	18	19	20	21
1. TAS-20 Difficulty Identifying Feelings	–																				
2. TAS-20 Difficulty Describing Feelings	0.77 ***	–																			
3. TAS-20 Externally Oriented Thinking	0.32 ***	0.44 ***	–																		
4. TAS-20 Total score	0.89 ***	0.90 ***	0.66 ***	–																	
5. PAQ Negative-difficulty identifying feelings	0.84 ***	0.77 ***	0.36 ***	0.82 ***	–																
6. PAQ Positive-difficulty identifying feelings	0.73 ***	0.68 ***	0.35 ***	0.73 ***	0.80 ***	–															
7. PAQ Negative-difficulty describing feelings	0.76 ***	0.85 ***	0.43 ***	0.83 ***	0.85 ***	0.72 ***	–														
8. PAQ Positive-difficulty describing feelings	0.68 ***	0.74 ***	0.40 ***	0.75 ***	0.74 ***	0.87 ***	0.80 ***	–													
9. PAQ General-externally orientated thinking	0.54 ***	0.65 ***	0.58 ***	0.71 ***	0.63 ***	0.58 ***	0.68 ***	0.64 ***	–												
10. PAQ Total score	0.78 ***	0.83 ***	0.51 ***	0.87 ***	0.89 ***	0.87 ***	0.91 ***	0.89 ***	0.85 ***	–											
11. DFS Total score	0.25 ***	0.20 ***	–0.09*	0.16 ***	0.18 ***	0.16 ***	0.16 ***	0.17 ***	0.00	0.13 ***	–										
12. SIPI Positive-Constructive Daydreaming	–0.08 *	–0.13 ***	–0.31 ***	–0.20 ***	–0.10 **	–0.13 ***	–0.15 ***	–0.14 ***	–0.26 ***	–0.19 ***	0.51 ***	–									
13. SIPI Guilt and Fear-of-Failure Daydreaming	0.42 ***	0.35 ***	0.08 *	0.37 ***	0.38 ***	0.38 ***	0.31 ***	0.34 ***	0.14 ***	0.32 ***	0.39 ***	0.20 ***	–								
14. SIPI Poor Attentional Control	0.36 ***	0.34 ***	0.06	0.33 ***	0.28 ***	0.24 ***	0.29 ***	0.25 ***	0.13 ***	0.26 ***	0.56 ***	0.14 ***	0.46 ***	–							
15. ERQ-F Fantasizing	0.14 ***	0.12 **	–0.04	0.10 *	0.12 **	0.13 ***	0.10 **	0.09 *	–0.03	0.08 *	0.56 ***	0.62 ***	0.39 ***	0.32 ***	–						
16. PERS-S Negative-activation	0.37 ***	0.35 ***	0.08 *	0.34 ***	0.30 ***	0.29 ***	0.30 ***	0.31 ***	0.14 ***	0.28 ***	0.33 ***	–0.01	0.43 ***	0.48 ***	0.21 ***	–					
17. PERS-S Negative-intensity	0.37 ***	0.29 ***	–0.03	0.28 ***	0.29 ***	0.25 ***	0.26 ***	0.25 ***	0.07	0.23 ***	0.34 ***	0.05	0.40 ***	0.46 ***	0.23 ***	0.76 ***	–				
18. PERS-S Negative-duration	0.43 ***	0.40 ***	0.07	0.39 ***	0.35 ***	0.31 ***	0.36 ***	0.35 ***	0.20 ***	0.34 ***	0.33 ***	–0.06	0.41 ***	0.50 ***	0.16 ***	0.79 ***	0.76 ***	–			
19. PERS-S Positive-activation	–0.30 ***	–0.36 ***	–0.24 ***	–0.36 ***	–0.29 ***	–0.36 ***	–0.31 ***	–0.40 ***	–0.36 ***	–0.39 ***	–0.07	0.26 ***	–0.09 *	–0.19 ***	0.10 **	–0.29 ***	–0.18 ***	–0.34 ***	–		
20. PERS-S Positive-intensity	–0.21 ***	–0.35 ***	–0.31 ***	–0.34 ***	–0.25 ***	–0.31 ***	–0.31 ***	–0.36 ***	–0.39 ***	–0.38 ***	–0.00	0.36 ***	–0.02	–0.15 ***	0.15 ***	–0.19 ***	–0.00	–0.23 ***	0.73 ***	–	
21. PERS-S Positive-duration	–0.36 ***	–0.40 ***	–0.27 ***	–0.42 ***	–0.34 ***	–0.39 ***	–0.37 ***	–0.43 ***	–0.34 ***	–0.42 ***	–0.14 ***	0.29 ***	–0.17 ***	–0.33 ***	0.07	–0.43 ***	–0.32 ***	–0.45 ***	0.75 ***	0.73 ***	–

*Note*. * *p* < 0.05; ** *p* < 0.01; *** *p* < 0.001.

**Table 3:** Full Measurement Set Exploratory Factor Analysis: Replication of Preece and Gross [25].

Measures	Factor 1	Factor 2	Factor 3	Factor 4	Factor 5	Uniqueness
TAS-20 DIF	**0.83**	0.15	0.06	0.02	–0.01	0.24
TAS-20 DDF	**0.67**	0.10	–0.04	0.05	0.24	0.23
TAS-20 EOT	0.06	–0.02	–0.06	–0.06	**0.66**	0.47
PAQ Negative-DIF	**0.95**	0.01	0.06	–0.01	–0.01	0.15
PAQ Positive-DIF	**0.93**	–0.07	–0.09	0.00	–0.12	0.21
PAQ Negative-DDF	**0.80**	0.03	0.03	–0.01	0.18	0.17
PAQ Positive-DDF	**0.84**	–0.04	–0.13	0.01	0.00	0.22
PAQ General-EOT	**0.42**	–0.07	–0.09	–0.05	**0.50**	0.31
DFS Total score	0.01	0.13	–0.15	**0.75**	–0.01	0.36
SIPI Positive-Constructive Daydreaming	–0.02	–0.17	0.13	**0.76**	–0.15	0.32
SIPI Guilt and Fear-of-Failure Daydreaming	0.27	**0.30**	0.06	**0.33**	0.01	0.60
SIPI Poor Attentional Control	0.01	**0.41**	–0.14	**0.39**	0.10	0.53
ERQ-Fantasizing	0.02	0.01	0.07	**0.77**	0.10	0.39
PERS-S Negative-activation	–0.04	**0.86**	–0.07	0.02	0.04	0.23
PERS-S Negative-intensity	0.06	**0.88**	0.10	–0.01	–0.08	0.24
PERS-S Negative-duration	0.03	**0.85**	–0.09	–0.02	0.02	0.21
PERS-S Positive-activation	–0.07	–0.01	**0.83**	0.01	0.06	0.28
PERS-S Positive-intensity	0.03	0.12	**0.89**	0.02	–0.09	0.21
PERS-S Positive-duration	–0.04	–0.20	**0.80**	0.01	0.02	0.21
Eigenvalues	7.44	3.23	2.14	1.30	0.81	–
The proportion of total variance for the rotated solution (%)	25.85	14.94	12.74	11.09	5.93	–

*Note*. Factor loadings ≥ 0.30 are in bold.

**Table 4:** Factor Intercorrelations for Stage 1 (Full Measurement Set) EFA Solution.

Factors	Factor 1	Factor 2	Factor 3	Factor 4	Factor 5
Factor 1	–				
Factor 2	0.38	–			
Factor 3	–0.38	–0.30	–		
Factor 4	0.11	0.28	0.14	–	
Factor 5	0.52	0.06	–0.33	–0.15	–

**Table 5:** Reduced Measurement Set Exploratory Factor Analysis (Excluding ERQ-F and PERS-S).

Measures	Factor 1	Factor 2	Factor 3	Factor 4	Uniqueness
TAS-20 DIF	**0.90**	0.01	0.02	–0.02	0.18
TAS-20 DDF	**0.62**	0.08	0.07	0.29	0.21
TAS-20 EOT	–0.01	–0.00	–0.02	**0.72**	0.49
PAQ Negative-DIF	**0.77**	0.21	–0.06	0.00	0.14
PAQ Positive-DIF	0.22	**0.77**	–0.01	–0.06	0.16
PAQ Negative-DDF	**0.57**	0.22	–0.00	0.23	0.18
PAQ Positive-DDF	–0.01	**0.89**	0.06	0.11	0.09
PAQ General-EOT	0.11	0.21	–0.02	**0.62**	0.30
DFS Total score	–0.05	0.06	**0.93**	0.00	0.14
SIPI Positive-Constructive Daydreaming	–0.11	0.07	**0.49**	**–0.31**	0.63
SIPI Guilt and Fear-of-Failure Daydreaming	**0.40**	0.02	**0.37**	–0.10	0.64
SIPI Poor Attentional Control	**0.36**	–0.19	**0.54**	0.09	0.56
Eigenvalues	6.01	2.15	0.83	0.72	–
The proportion of total variance for the rotated solution (%)	25.80	17.13	13.58	12.47	–

*Note.* Factor loadings ≥ 0.30 are in bold.

**Table 6:** Factor Intercorrelations for Stage 2 (Reduced Measurement Set) EFA Solution.

Factors	Factor 1	Factor 2	Factor 3	Factor 4
Factor 1	–			
Factor 2	0.79	–		
Factor 3	0.26	0.13	–	
Factor 4	0.53	0.55	–0.11	–

## Data Availability

De-identified data are available from the corresponding author upon reasonable request.
